# Reduced palmitic acid content in soybean as a result of mutation in *FATB1a*

**DOI:** 10.1371/journal.pone.0262327

**Published:** 2022-03-10

**Authors:** Militza Carrero-Colón, Karen Hudson

**Affiliations:** Crop Production and Pest Control Research Unit, USDA-Agricultural Research Service, West Lafayette, Indiana, United States of America; University of Guelph, CANADA

## Abstract

The fatty acid component of commodity soybean seeds typically consists of approximately 12–15% saturated fatty acids in the form of palmitic acid and stearic acid. An important goal in soybean breeding is the reduction of saturated fats, in order to produce healthier vegetable oils for food applications. Genetic approaches have been instrumental in reducing levels of palmitic acid, which is the most abundant saturated fat in soybean seeds. In this study we describe a new mutant allele of the *FATB1a* gene that encodes a palmitoyl-acyl carrier protein thioesterase. The mutation is expected to result in early termination of the FATB1A protein and mutant seeds carrying this allele contain 5.5% palmitic acid. This new allele can be introduced into conventional soybean lines, alone or in combination with other modifications to generate soybean lines with improved oil composition.

## Introduction

Soybean (*Glycine max*) is an oilseed crop of global importance, and soybean oil comprises almost a third of vegetable oils consumed around the world [1]. Genetic approaches have been used to modify all aspects of the soybean oil profile (reviewed in [2–4]). Of particular emphasis in the improvement of soybean oil is the reduction in levels of saturated fatty acids, such as stearic acid and palmitic acid. High intake of dietary palmitic acid is believed to contribute to increased cholesterol levels and increased risk of heart disease in humans [5, 6]. Commodity soybean oil has relatively low levels of palmitic acid, around 10–12%, but reductions in this level are genetically tractable, therefore 7% total saturated fats (stearic acid and palmitic acid combined) is a goal for breeding for oil composition [4]. Specifically, it has been demonstrated that mutation in the *KASIII* or *FATB1a* genes can result in reduced levels of palmitic acid. Mutations in *KASIII* were initially isolated as the *fap1* locus, represented by a single nucleotide change that results in a premature truncation of the KASIII protein and reduces palmitic acid levels by 20–30% [7]. The *fap3* locus corresponds to the *FATB1a* gene, and several independent deletions and single nucleotide polymorphisms have been identified in this gene that reduce palmitic acid content to as little as 6% [8–13] similar to the effect of silencing the expression of *FATB* gene in soybean seeds [14]. Mutations in both *KASIII* and *FATB1a* can function additively to reduce palmitic acid content in seeds [7]. The *FATB1a* gene encodes an acyl-acyl carrier protein thioesterase which controls export of the 16-carbon fatty acids from the plastid after synthesis, and therefore influences the balance of saturated to unsaturated fatty acids in the seed [15]. As further developments are made to produce healthier soybean oil low in *trans* fats, with a high oleic profile that finds broader markets and new uses, continued efforts to identify and deploy non-transgenic variation to reduce saturated fats will be required. In this work we describe the isolation and characterization of a new nonsense allele of *FATB1a* that can be a tool to develop improved soybean germplasm.

## Materials and methods

The mutant population was generated by NMU treatment and an ongoing screen of fatty acid profiles in approximately 5,000 M_3_ bulk seed samples was performed as described previously [16]. For characterization of the low palmitic acid trait, plants were harvested in bulk from 1.8 meter field plot rows in West Lafayette, Indiana during the 2016–2020 growing seasons (excluding 2019), and individual seeds from multiple individuals were phenotyped. Ten single seeds were chipped for analysis by GC as described previously [12]. Statistical significance of fatty acid content was performed by comparing wild type and mutant composition each year using two-tailed, type 2 *t*-tests.

In the field in 2015 the putative mutant line (known as plant 19479) was outcrossed to the purple flowered cultivar Prize (PI 548554), to facilitate genetic mapping of the low palmitic acid trait, and the F_1_ plant from this cross was grown to maturity in the field during the 2016 growing season. To test for complementation of the new mutation to a previously identified *FATB1a* mutant, plant 19479 was crossed in the field during 2017 to a line carrying a reference mutant allele of *FATB1a* (*FATB1a*_*G180D*_) [12], and the F_1_ plants were germinated and grown to maturity in the greenhouse in 16h light/8 hour dark cycles at a temperature of 27C. For both crosses F_2_ seeds were harvested and subjected to single seed gas chromatograph phenotyping to measure fatty acid content in parallel with single seed genotyping as described previously [17]. For the complementation cross, 49 single seeds were analyzed, and for the backcross population, 80 single seeds were analyzed.

Primers for amplification and sequencing of the *FATB1a* gene were 5’-GTCTTCTGGTGGCTTGAAGG-3’ and 5’-CCCAGACAAATTTCCAAAGC-3’ for the first and second exons, and 5’-GACATAGTTCAAGTGGACACT-3’ and 5’-TTCACAACACCAAAGTTGTTCAC-3’ to cover exons three to six. PCR amplification was performed using an initial 60 second denaturation at 95C, followed by 5 cycles of 94C for 30 seconds, 56C for 20 seconds, and 68C for 4 minutes, followed by 20 cycles of 94C for 30 seconds, 58C for 20 seconds and 68C for 4 minutes. PCR amplicons were sequenced using the same primers and dye terminator sequencing and BigDye (Life Technologies, Waltham, MA) standard kit protocols. Genotyping for the *FATB1a*_*Q52STOP*_ polymorphism was performed with a cleaved amplified polymorphic sequence (CAPS) marker designed to recognize the polymorphism. DNA was extracted from seed chips using the Mega EZ Plant 96-well DNA kit. Genotyping primer sequences were 5’- AACTGATGTGCTGTGCTGTT-3’ and 5’- TCAGCGGCCAAGAAAATTGT-3’. PCR and digestion reaction conditions were 1 minute at 95C, 30 cycles of 94C for 20 seconds, 25 seconds at 58C, 68C for 1 minute, and a final extension for 7 minutes at 68C. PCR amplicons were digested overnight at 37C with the restriction enzyme *HhaI* (New England Biolabs) which cuts the wild type sequence, and visualized by electrophoresis on 1% TBE agarose gel.

## Results and discussion

A line (referred to as plant 19479) with low levels of palmitic acid was identified from an ongoing mutant screen for altered fatty acid composition in the Williams-82 cultivar. Based on the uniform low levels of palmitic acid in seeds from self-pollinated individual M_3_ plants, it was inferred that this line was homozygous. We observed that levels of palmitic acid were reproducibly and statistically significantly low, ranging from 5.9% to 6.4% over four seasons in the field, nearly a 50% reduction in palmitic acid (Table 1). We also observed statistically significant increases in oleic acid, particularly in 2016–2018, however the extent of increase in oleic acid levels was not consistent for all seasons examined.

**Table 1:** Fatty acid profiles of *FATB1a*_*Q52STOP*_ mutants and wild type Williams-82 field grown seed.

Year	Genotype	Palmitic Acid	Stearic Acid	Oleic Acid	Linoleic Acid	Linolenic Acid
2016	W-82	11.6±0.4	3.7±0.2	23.0±2.4	55.5±2.1	6.3±0.3
	*Q52STOP*	5.9±0.4***	3.7±0.6	38.0±2.9***	47.0±3.5***	5.5±0.3***
2017	W-82	11.0±0.6	4.3±0.2	23.9±2.8	53.2±2.2	7.6±0.4
	*Q52STOP*	6.4±0.4***	3.5±0.3***	33.3±4.2***	49.1±3.8	7.7±0.5
2018	W-82	11.6±0.4	4.0±0.2	21.2±1.6	56.5±1.2	6.8±0.6
	*Q52STOP*	6.1±0.3***	3.7±0.7	34.1±5.2***	50.1±5.1	5.9±0.5
2020	W-82	11.8±0.3	3.8±0.1	21.2±0.7	56.1±0.8	7.2±0.5
	*Q52STOP*	6.3±0.2***	4.0±0.4	23.3±1.1***	57.4±0.9	9.1±0.6***

*** indicates significance level of *p*<0.001 in Student’s *t*-test.

Values are means of ten samples plus/minus standard deviation.

**Fig 1:**
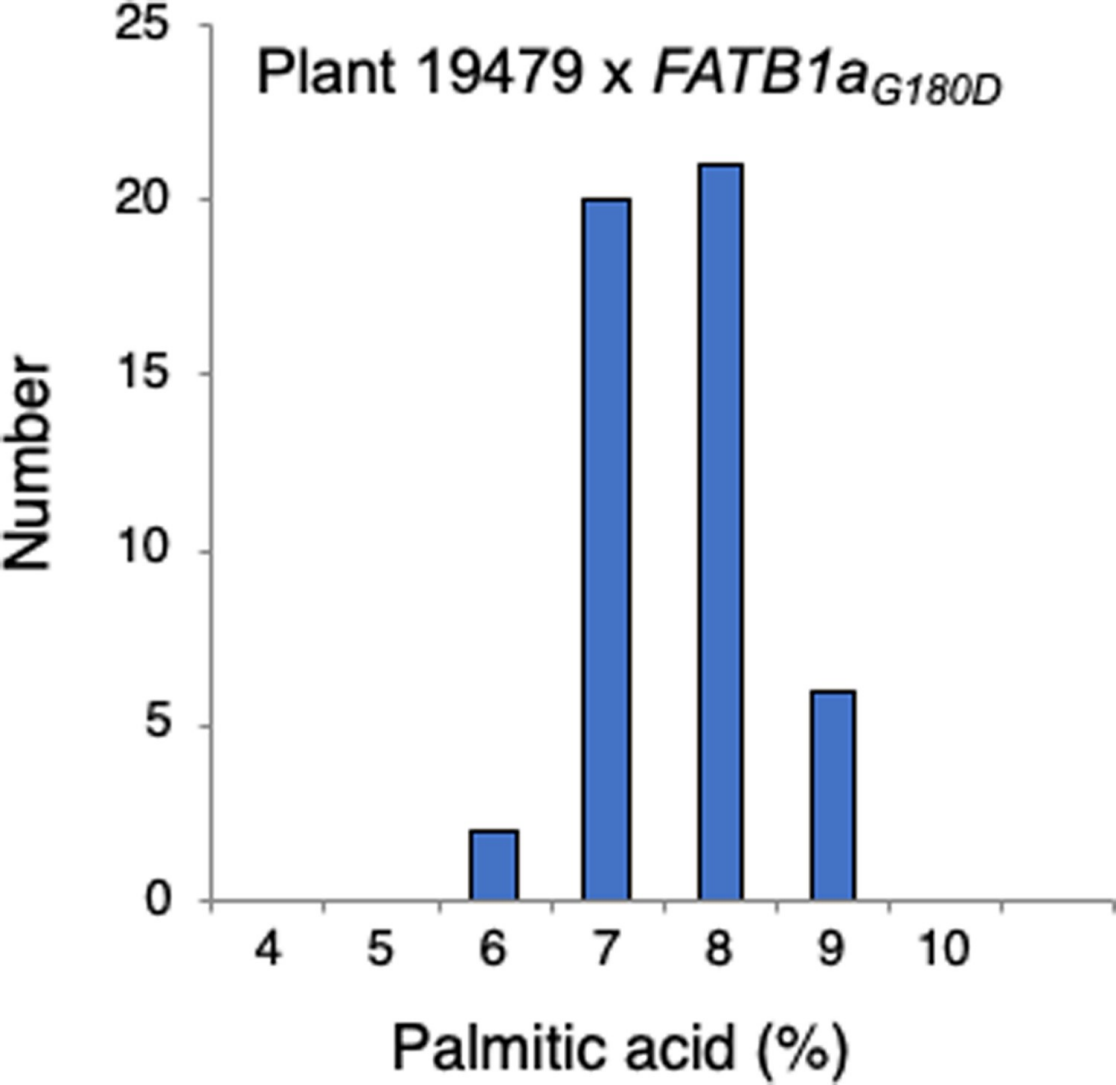
Palmitic acid content in F_2_ seeds from complementation test cross to *FATB1a*_*G180D*_. Fatty acid content was measured in 49 F_2_ individual seeds.

To determine if this mutant represented a new or previously characterized locus affecting palmitic acid content, plant 19479 was crossed to a line carrying a known allele of *FATB1a* (*FATB1a*_*G180D*_*)* in which seeds typically contain 6.8% palmitic acid [12]. The F_1_ plant was grown to maturity and fatty acid composition was measured in individual F_2_ seeds. No wild type individuals were identified, and all individuals had low palmitic acid levels below 9%, which suggested that the lesion in the low palmitic line did not complement the reference mutation in *FATB1a* (Fig 1).

**Fig 2:**
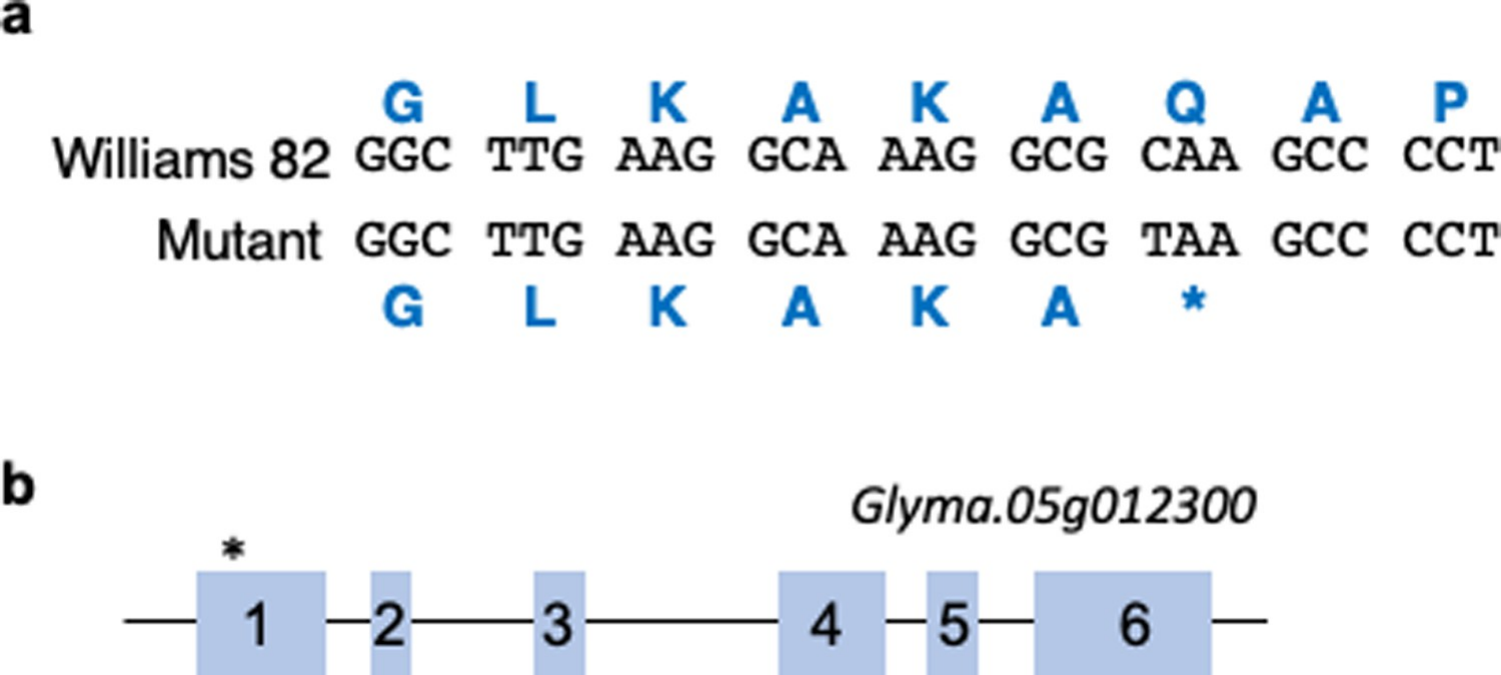
Polymorphism in *FATB1a*. a. DNA and predicted amino acid sequence (blue) of the *FATB1a* gene (Glyma.05g012300) in Williams 82 soybean and the mutant. b. Early termination signal (*) occurs within the first exon of the predicted protein.

The *FATB1a* gene (Glyma.05g012300, version Glyma2.0) was sequenced as a candidate for causing the low palmitic acid phenotype. A single nucleotide polymorphism (C to T) consistent with NMU mutagenesis was identified at base position 154 in the predicted *FATB1a* transcript, which resulted in the introduction of an early termination signal in place of amino acid Q52 (Fig 2A). The full-length protein is 417 amino acids long, and this mutation is expected to result in a truncation early in the first exon (Fig 2B). We will refer to this allele henceforth as *FATB1a*_*Q52STOP*_.

**Fig 3:**
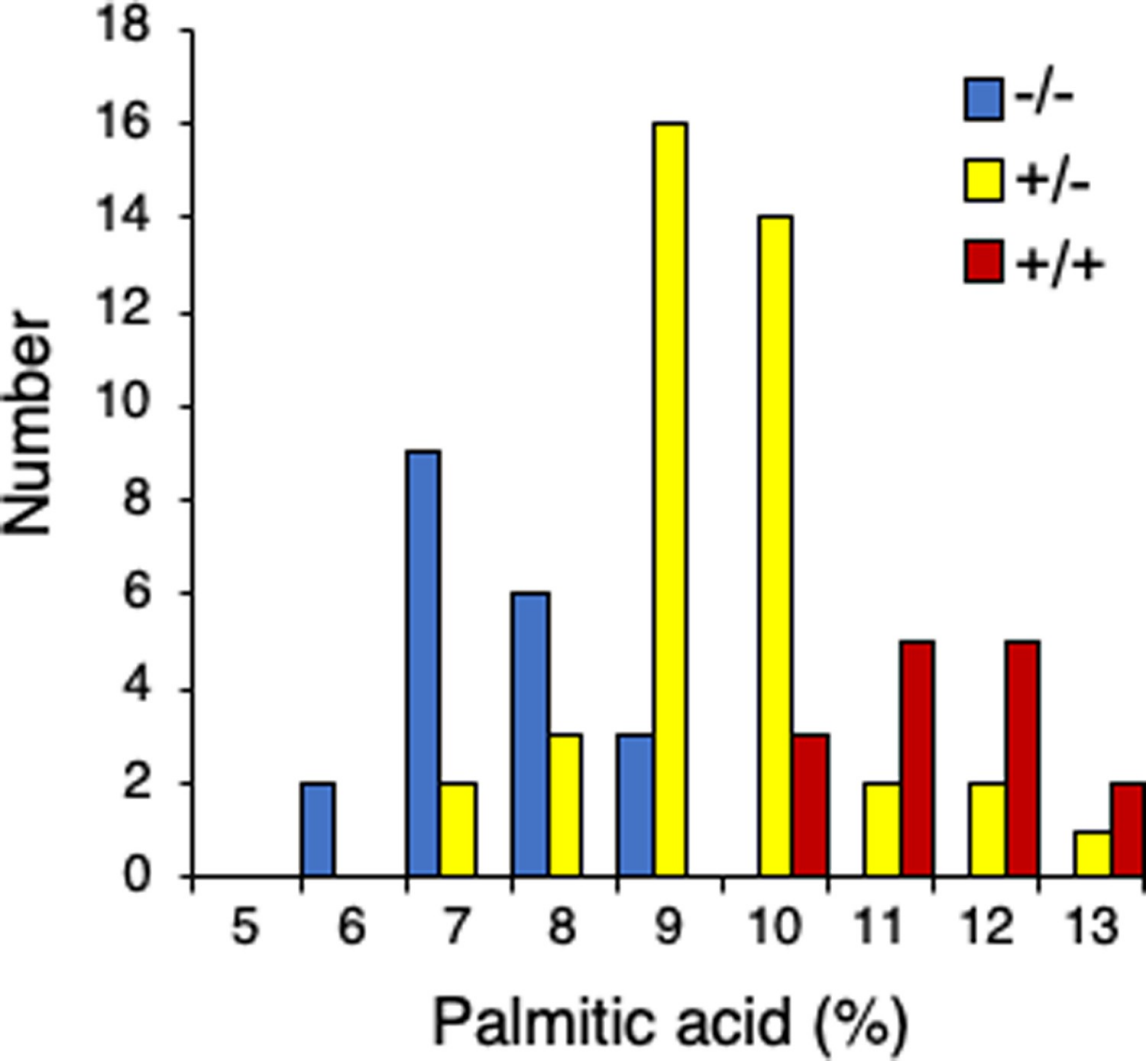
Co-segregation of *FATB1a*_*Q52STOP*_ and low palmitic acid phenotype. Distribution of palmitic acid content and genotype for 80 F_2_ individuals from an outcross. (-/-) homozygous for *FATB1a*_*Q52STOP*_, (+/-) heterozygous for *FATB1a*_*Q52STOP*_, (+/+) wild type *FATB1a*.

We took advantage of the fact that the polymorphism caused the loss of a *HhaI* restriction site in the mutant to design a codominant polymorphic marker to genotype for the presence of the *FATB1a*_*Q52STOP*_ mutation. To determine if the mutation co-segregated with the low palmitic acid phenotype, the mutant line was crossed to the cultivar Prize and individual F_2_ seeds were chipped for fatty acid phenotyping and genotyped. Palmitic acid values in individuals seeds in the F_2_ population ranged from 5.6% to 16.8% palmitic acid. The *FATB1a*_*Q52STOP*_ mutation co-segregated with the low palmitic acid phenotype, and thus is likely causative (Fig 3). Homozygous mutant individuals ranged from 5.6 to 8.6% palmitic acid with a mean of 6.9%, which was slightly higher than the levels observed in field grown plant populations, and may be a result of differences between the field and greenhouse environments. Heterozygous individuals averaged 9.4% palmitic acid. Wild type individuals contained 11.3% palmitic acid, which was similar to the levels observed in field-grown seeds.

The *FATB1a*_*Q52STOP*_ polymorphism results in a low level of seed palmitic acid, comparable to the low palmitic acid *fap3* mutants previously described in soybean [8, 10, 12, 18, 19]. This mutation represents an early termination of the FATB1A protein. We observed in some years a significant increase in oleic acid levels in F*ATB1a*_*Q52STOP*_ mutants, which has been observed in previous studies of *fap3* mutants [19, 20], however this was not consistent for all years in this study, and elevated oleic acid levels were not associated with the *FATB1a*_*Q52STOP*_ genotype within the segregating population, and therefore may be a result of secondary mutations in this line. No agronomic or physiological abnormalities were observed in the *FATB1a*_*Q52STOP*_ plants; however, yield was not directly tested in large scale field experiments. Reduction in palmitic acid levels or mutation in *KASIII* has previously been associated with negative effects on seed yield [20, 21]. Further studies will determine the extent to which this allele can be used by breeders to reduce levels of palmitic acid to develop healthier soybean oils for the edible oil market.

The single nucleotide polymorphism in *FATB1a* can be readily followed with a PCR-based genotyping marker to facilitate introgression into elite germplasm. This stable and non-transgenic mutation can be used in the development of conventional soybean lines with reduced saturated fat content, alone or in combination with other seed composition traits.
